# Supplementary material for: From sequence to enzyme mechanism using multi-label machine learning
Source: BMC Bioinformatics. 2014 May 19;15:150. doi: 10.1186/1471-2105-15-150 (PMC4229970; doi:10.1186/1471-2105-15-150)
Supplement: Additional file 2 — Java code of ml2db. Additional file ml2db_code.tar.gz contains the Java source code to run the multi-label machine learning experiments and save the results to database. The code’s Javadoc is included. [file 1471-2105-15-150-S2.zip › additional file 2/ml2db/ml2db/doc/main/ml2db/Serializer.html]

Serializer


JavaScript is disabled on your browser.


- Overview
- Package
- Class
- Use
- Tree
- Deprecated
- Index
- Help

- Prev Class
- Next Class

- Frames
- No Frames

- All Classes

- Summary:
- Nested |
- Field |
- Constr |
- Method

- Detail:
- Field |
- Constr |
- Method


main.ml2db

## Class Serializer

- java.lang.Object
- - main.ml2db.Serializer

- Direct Known Subclasses:
  :   MulanSerializer

  ---

    

  ```
  public abstract class Serializer
  extends java.lang.Object
  ```

  Class

  Version:
  :   27 Jul 2011

  Author:
  :   Luna De Ferrari ldf(at)st-andrews.ac.uk

- - ### Constructor Summary

    Constructors

    Constructor and Description

    `Serializer()`
  - ### Method Summary

    Methods

    | Modifier and Type | Method and Description |
    | --- | --- |
    | `static java.lang.Object` | `clone(java.io.Serializable in)` To test where in a class an instance of Object is created, hence making serialization impossible. |
    | `java.lang.String` | `getBaseFileName()` |
    | `java.lang.String` | `getDirectoryForSerializedObject()` |
    | `java.lang.String` | `getFileForSerializedObject()` |
    | `java.io.File` | `getLogFile()` |
    | `static void` | `main(java.lang.String[] args)` |
    | `void` | `serialize()` |
    | `void` | `setFileForSerializedModel()` |
    | `void` | `setLogFile()` |

    - ### Methods inherited from class java.lang.Object

      `equals, getClass, hashCode, notify, notifyAll, toString, wait, wait, wait`

- - ### Constructor Detail


    - #### Serializer

      ```
      public Serializer()
      ```
  - ### Method Detail


    - #### getBaseFileName

      ```
      public java.lang.String getBaseFileName()
      ```

      Returns:
      :   m\_baseFileName


    - #### getDirectoryForSerializedObject

      ```
      public java.lang.String getDirectoryForSerializedObject()
      ```


    - #### getFileForSerializedObject

      ```
      public java.lang.String getFileForSerializedObject()
      ```


    - #### getLogFile

      ```
      public java.io.File getLogFile()
      ```


    - #### serialize

      ```
      public void serialize()
      ```


    - #### setFileForSerializedModel

      ```
      public void setFileForSerializedModel()
      ```


    - #### setLogFile

      ```
      public void setLogFile()
      ```


    - #### clone

      ```
      public static final java.lang.Object clone(java.io.Serializable in)
      ```

      To test where in a class an instance of Object is created, hence making
      serialization impossible.

      Parameters:
      :   `in` -

      Returns:


    - #### main

      ```
      public static void main(java.lang.String[] args)
      ```


- Overview
- Package
- Class
- Use
- Tree
- Deprecated
- Index
- Help

- Prev Class
- Next Class

- Frames
- No Frames

- All Classes

- Summary:
- Nested |
- Field |
- Constr |
- Method

- Detail:
- Field |
- Constr |
- Method
